# Parents don't dictate the menu: Lack of strong host‐specific parental effects may help maintain dietary generalism

**DOI:** 10.1002/ecy.70499

**Published:** 2026-10-06

**Authors:** Emma J. Sellers, Mayra C. Vidal, Dhaval K. Vyas, Mykaela Tanino‐Springsteen, Gina M. Wimp, Mattheau S. Comerford, Mariana Abarca, Shannon M. Murphy

**Affiliations:** ^1^ Department of Biological Sciences University of Denver Denver Colorado USA; ^2^ Department of Biology University of Massachusetts Boston Boston Massachusetts USA; ^3^ Department of Biology Georgetown University Washington DC USA; ^4^ Department of Biology University of Pennsylvania Philadelphia Pennsylvania USA; ^5^ Department of Biological Sciences Smith College Northampton Massachusetts USA; ^6^ Present address: Department of Animal Ecology Netherlands Institute of Ecology (NIOO‐KNAW) Wageningen The Netherlands

**Keywords:** Erebidae, fall webworm (*Hyphantria cunea*), fitness, host plant, Lepidoptera, transgenerational effect

## Abstract

The environment or experiences of a parent generation can impact the fitness of the next generation, a phenomenon known as parental effects. While a high‐quality diet can directly benefit an individual and a low‐quality diet can reduce fitness, a parental effect occurs only when there is a transgenerational response to these stimuli that manifests in the individual's offspring. In dietary generalists, a parental effect could play an important role for offspring performance if the species experiences considerable variation in quality across host plants; positive parental effects may result from host matching between parents and offspring on high‐quality host plants, whereas negative effects may result from host matching on low‐quality host plants or parent‐offspring host mismatching. A lack of strong parental effects across host plants may also help explain the maintenance of dietary generalism for generalist herbivores. We investigated whether parental effects occur in the generalist herbivore fall webworm (*Hyphantria cunea*) by testing whether offspring performance depended on the host plant on which the parental generation was reared. We found that parental effects do not play an important role for fall webworm and the weak effects that do exist depend on both the larval and parental host plant. Rather than demonstrating benefits of parent‐host matching, our results demonstrate that the offspring of parents from one poor‐quality host performed worse on one high‐quality host than offspring from any of the other hosts. Overall, our study suggests that larval host plant identity rather than parental effects is important to the performance of this generalist herbivore. Notably, a lack of strong, consistent parental effects may help explain the maintenance of dietary generalism in an extreme generalist herbivore.

## INTRODUCTION

Offspring survival and fitness can be dependent on the experiences and decisions of the previous generation. A parental effect occurs when there is a transgenerational response to environmental stimuli, such as diet quality, that manifests in an organism's offspring (Mousseau & Fox, 1998). Environmental conditions experienced by parents can influence offspring traits such as development time, growth rate, diapause induction, polyphenisms, and the production of sexual forms (Fox & Mousseau, 1998; Mousseau & Dingle, 1991). Parental effects have been described across taxa, including annelides (Guillaume et al., 2016), nematodes (Dey et al., 2016), fish (Reznick et al., 1996; Vega‐Trejo et al., 2018), and insects (Fox & Mousseau, 1998). In herbivorous insects, female parents can affect offspring phenotype and survival through oviposition choices (Woestmann & Saastamoinen, 2016) and egg nutrient allocation (Fox & Mousseau, 1998). Furthermore, the environment that parents experience can affect the viability and establishment of their offspring (van Asch et al., 2010; Mosseau and Fox, 1998). For example, the “silver‐spoon effect” suggests that offspring often reap an additional transgenerational benefit of matching the same favorable environment that the female parent experienced (i.e., parent‐offspring matching; Uller et al., 2013). However, not all parents experience optimal environments.

Some insects have evolved adaptive maternal effects such that a female parent in a poor or stressful environment can provision her offspring so that they will have increased fitness if they experience the same poor environment (Rotem et al., 2003; Yin et al., 2019). For example, if a female parent experiences a stressful environment, such as a low‐quality diet, she may reduce egg cluster size while increasing the nutrients provisioned to each egg, so that her offspring can better succeed in that same stressful environment (Rotem et al., 2003). However, not all parents can provide conditions to assist offspring survival on a low‐quality diet. For example, in the Indianmeal moth (*Plodia interpunctella*), offspring from parents fed a low‐quality diet were smaller and had poor immune function compared to offspring from parents fed a high‐quality diet (Triggs & Knell, 2012), and when winter moth (*Operophtera brumata*) parents fed on older leaves, the development time of their offspring was increased (van Asch et al., 2010). These findings may explain why we find parent‐offspring mismatch for larvae feeding on poor‐quality host plants (Petrén et al., 2021). Hence, offspring from parents fed on a low‐quality diet could be either: (1) well equipped to thrive on the same low‐quality diet due to parental provisioning that enhances their ability to cope with the low‐quality diet (an adaptive maternal effect), or (2) poorly equipped to survive in any environment because they did not receive enough resources from parents, and this limits their overall viability. Herbivorous insect species with a broad diet range provide unique opportunities to examine whether the parents' diet yields transgenerational benefits to their offspring.

The importance of parental effects on offspring fitness may vary with diet breadth, a key life history trait. In dietary specialists, where both parents and offspring are likely to encounter the same limited set of host plants, parental effects are more likely to be adaptive because the offspring's future environment is predictable (parent‐offspring host matching) and positive effects of host plant matching may help explain the evolution of dietary specialization. For example, Cahenzli et al. (2015) found that while cabbage white (*Pieris rapae*) larvae performed well on high‐quality hosts regardless of parental diet, those whose parents fed on low‐quality hosts performed better on low‐quality plants than offspring from high‐quality‐fed parents (but see Petrén et al., 2021, which found no pattern with *Pieris rapae*). Conversely, in generalist herbivores that feed on a broad range of hosts, such predictability is reduced and host plants are more likely to be mismatched between generations, which may weaken or preclude selection for parental effects. However, even in herbivores with more generalized diets, host plant quality can still mediate transgenerational effects. For instance, in the polyphagous moth *Spodoptera littoralis*, parental host plant had no effect on oviposition or larval host choice, but larval performance was higher on high‐quality hosts when parents also fed on high‐quality hosts (Rösvik et al., 2020). Additionally, parental effects might be found in herbivores with a broader diet because diet specialization could still happen at the local population level. Dietary generalism for an herbivore species could be a trait realized only at the species level across a wide geographic range instead of at the population level (Fox & Morrow, 1981). If generalist species are composed of multiple specialized populations, generalists may feed consistently across generations on the same host, thereby increasing the potential benefit of parental provisioning in a stressful environment if parents can anticipate future environments and an adaptive parental effect exists.

We investigated parental effects in fall webworm (*Hyphantria cunea*, hereafter FW), which is an extreme dietary generalist species of moth, feeding on over 600 plant species worldwide (Schowalter & Ring, 2017; Warren & Tádic, 1970). While FW is a dietary generalist as a species, individual larvae are functionally monophagous as they are isolated on the plant on which their female parent lays her eggs. Female FW moths are reported to rarely disperse far from eclosion sites (Suzuki & Kunimi, 1981), so they potentially oviposit on the same host plant on which they developed as larvae. As FW larvae feed on a single plant that may be the same plant upon which the female parent fed, this makes FW an ideal study system to test for parental effects in a dietary generalist. Morris (1967) suggested that when a parental generation of FW was reared on an intermediate quality host plant, the next generation experienced severe mortality even when reared on high‐quality foliage. However, Morris (1967) did not rear larvae on the same host plant as the parental generation and had several confounding variables that obscured results. Decades of research with FW from different populations across locations has shown that larval performance varies considerably on their local host plant species (Warren & Tádic, 1970; Greenblatt et al., 1978; Mason et al., 2011; Jang et al., 2015; Murphy & Loewy, 2015; Vidal & Murphy, 2018; Vyas & Murphy, 2022, unpublished data). Thus, the host plant on which a FW female parent lays her egg cluster can have repercussions for the survival and performance of her offspring; however, whether there are long‐lasting fitness consequences for the offspring and future generations is unknown.

The offspring of a generalist herbivore, such as FW, have the potential to experience the same environment as their parent (parent‐offspring host matching) or a different environment (parent‐offspring host mismatching; Figure 1). Offspring may encounter environments composed of host plants of different qualities, and parental effects could influence offspring performance in different ways. First, FW parents that experience favorable conditions, such as a high‐quality host plant, may produce larvae that perform worse when they feed on any other plant compared to if they were to feed on that same high‐quality plant. Thus, there may be a positive effect of environment matching between parental and larval host plants in which larvae always do best on their parental host independent of host plant quality (parent‐offspring host matching). Second, if FW parents feed on a low‐quality host plant, they may produce larvae that perform better if they feed on any plant other than the same low‐quality plant as their parents (parent‐offspring host mismatching). Additionally, if feeding on a low‐quality host plant translates into poor offspring performance, then the offspring of parents from low‐quality hosts would benefit from environmental mis‐matching between parental and larval hosts. Lastly, a generalist herbivore may not show any pattern of parental effects or mixed patterns of parental effects. For example, when reared on low‐quality host plants, parental plasticity, such as changes in feeding behavior or developmental rate, may partially buffer nutrient limitations. These compensatory mechanisms could reduce the extent to which parental environment influences offspring performance, dampening the expression of transgenerational effects (Logan et al., 2019).

**FIGURE 1:**
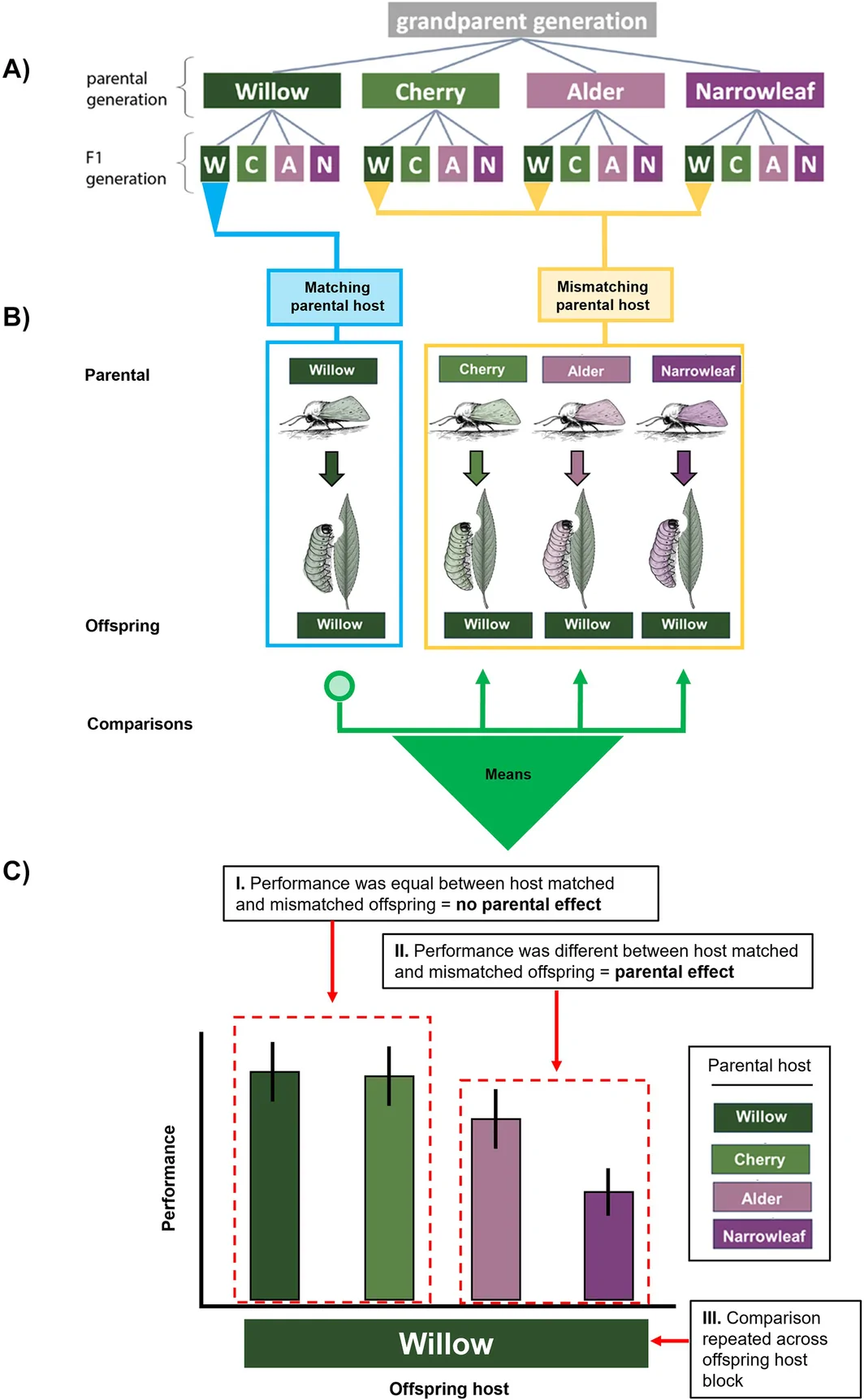
Overview of experimental design and data analysis. (A) Individuals in the parental generation were assigned to one of four host plants, mated within host plant treatment, and their offspring were divided into four groups, one for each host. Thus, (B) three groups of offspring per egg mass mismatched the parental host (yellow; only willow offspring shown) and one matched it (blue). (C) We analyzed the data by comparing means among parental host treatments, with no differences indicating the absence (I) and significant differences indicating the occurrence (II) of parental effects. Comparisons were performed separately for each offspring host (III). All moth, larva, and leaf illustrations were created by Mattheau S. Comerford.

Here, we addressed two research questions. First, does a generalist insect herbivore show parental effects in response to host plants of different qualities? Second, if we find evidence for parental effects, do they manifest when parents and offspring match or mismatch parental host plants? To answer these questions, we designed a full‐factorial, split‐brood experiment to test for dietary parental effects in FW. We reared FW on one of four host plants, two of which are high quality and two of which are low quality. These individuals formed our parental generation (Figure 1A); we split their offspring into four host plant treatment groups, and each group was reared either on the parental host or on one of the other three host plants. Our split‐brood design allowed us to test if FW parental or larval host is most important in determining fitness, which has important implications for understanding how generalists are able to perform well on different host plant species. With our experimental design, we were able to compare within larval hosts the performance of offspring from parents that had fed on different hosts to test if offspring perform differently depending on their parents' host plant. Additionally, when comparing within parental hosts, we can assess if larvae perform better when they are fed on the same host as their parent (parent‐offspring host matching) or different host (parent‐offspring host mismatching).

## METHODS AND MATERIALS

### Study system

FW is a species of moth widely found in North America and is an invasive species in some regions of Europe and Asia (Gomi & Takeda, 1996; Yang et al., 2006). There are two FW biotypes that represent distinct genetic lineages: the black‐ and the red‐headed biotypes (Vidal et al., 2019). Colorado contains an allopatric population of red‐headed FW, which is the biotype we used in our study. Red‐headed female moths typically mate soon after eclosion and usually lay a single egg cluster of over 100 eggs before dying (Loewy et al., 2013; Sellers, Hicks, et al., 2026).

To examine the parental effects of diet on the subsequent generation, we fed FW larvae two high‐quality plant species in Colorado, willow (*Salix* spp., likely crack willow *Salix fragilis*, Salicaceae, hereafter referred to as willow), and chokecherry (*Prunus virginiana*, Rosaceae, hereafter cherry), as well as two plant species that have been considered low‐quality host plants for FW in Colorado, thinleaf alder (*Alnus tenuifolia*, Betulaceae, hereafter alder), and narrowleaf cottonwood (*Populus angustifolia*, Salicaceae, hereafter narrowleaf). We define plant quality based on FW performance on these plants in previous experiments (for larvae on high‐quality plants have greater pupal mass, faster development, and higher survival than larvae on low‐quality plants; Murphy & Loewy, 2015; Vidal et al., 2020; Murphy unpublished data). In summer 2020, we collected hundreds of FW larvae from field sites along the Front Range of Colorado; these larvae were collected from a variety of host plants, including the four plants used in our experiment. These larvae completed their development in the lab on their natal host plant species, overwintered as pupae, eclosed as moths in spring 2021, and mated haphazardly across host plants; the offspring of these moths formed the parental generation for our study (Figure 1A). All larvae in our experiments were reared on one host plant species for the entire duration of their development until pupation, as larvae would do in the wild, but using leaves from different plant individuals to ensure any effects are not due to a specific plant genotype.

### Experimental design

During the summer of 2021, we reared FW larvae (source of population explained above) as part of a split‐brood design on one of the four host plant species introduced above: willow, cherry, alder, or narrowleaf (Figure 1A). These larvae pupated in fall 2021 and eclosed as moths in spring 2022. We mated these moths across matrilines (following methods outlined in Robinson‐Castillo et al., 2021) and ensured that for each mating, unrelated males and females were from the same host plant treatment (e.g., a female moth from a matriline reared on cherry would be mated to a male moth from a different matriline that also fed on cherry). We aimed to have nine matrilines for each host plant treatment (9 matrilines × 4 host plants = 36 matrilines); however, cherry and narrowleaf each had an extra matriline (*n* = 10 matrilines). The larvae produced from these matings were used for our experiment in 2022 (F1 generation; Figure 1A). Approximately 14 days after an egg cluster was laid, when head capsules were visible and before eggs hatched, we divided each cluster of eggs into four host plant treatment groups and placed each group in a 0.5‐L deli container provisioned with a leaf from one of the four host plants (Figure 1A). Thus, we had a total of 38 matrilines, each divided into four host plant treatment groups with each group feeding on one of the four host plants (*n* = 38 × 4 = total of 152 egg clusters). These 38 maternal lines represent our effective sample size, as the sibling larvae are not completely independent from each other.

We reared larvae using methods from Loewy et al. (2013) and Robinson‐Castillo et al. (2021). Briefly, we reared larvae on their assigned host plant until they pupated or died. At least twice a week, we removed old leaves and frass from the larval containers and added fresh leaves that we collected by clipping leaves from plants growing wild in the field; most larvae migrated to the new leaves and larvae on old leaves were gently transferred to the new leaves. To minimize mortality from handling, we reared larvae with their siblings in their host plant treatment group until they were about 2 weeks old, after which we haphazardly selected 15 individuals from each host plant treatment group to include in our experiment; at this point, we reared larvae in densities of five larvae per rearing container and separated them into new containers as necessary to avoid crowding, with larvae eventually being reared in containers of 2–3 larvae. While we aimed for 15 larvae in each treatment group, some groups ultimately had fewer larvae due to death or small egg cluster size (Appendix S1: Table S1). We reared a total of 2144 larvae across all maternal lines and treatments, but the 38 maternal lines represent our effective sample size (Appendix S1: Table S1). We reared all larvae in our laboratory at ambient temperature (room temperature = ~21°C) and exposed to natural daylight. After larvae pupated, we sexed the pupae (following methods in Loewy et al., 2013) and weighed them 30 days (±2 days) after pupation (to the nearest 0.01 mg; Mettler‐Toledo XP6, Columbus, Ohio).

We recorded three larval performance measures: survival (proportion), development time (days), and pupal mass (mg). Larval survival is a critical measure of fitness as an individual cannot reproduce if it dies before it reaches reproductive maturity. We measured proportion survival as individuals who survived to pupate out of the total number of individuals in that treatment group. Development time, measured as the number of days from hatch to pupation, is important for larvae in the field because those that develop faster may escape natural enemies and reach reproductive maturity more quickly (Price et al., 2011). Pupal mass, for both sexes, is a strong predictor of lifetime fitness and survival in most Lepidoptera (Bonsignore et al., 2019; Iyengar & Eisner, 1999; Jahant‐Miller et al., 2022). The effect of pupal mass on fitness has been demonstrated within FW as higher pupal mass in females is correlated with higher fecundity later in life (Loewy et al., 2013) and higher pupal mass in males is correlated with higher overwinter survival, increased eclosion, and longer longevity (unpublished data).

### Statistical analysis

To test how the parental host influenced the performance of offspring on different hosts, we used linear mixed models (LMMs) where the fixed effect was the interaction between the predictor variables of parental host and offspring host, and the random effect was matriline (Sellers, Tanino‐Springsteen, et al., 2026). We built three models, one for each performance metric as a response variable (pupal mass, survival, and development time). For pupal mass, the data followed a normal distribution, and we also included the sex (male or female) as another fixed effect. We treated survival as a binary response (survived or died), and since these data follow a binomial distribution of errors, we used generalized linear mixed models with the family = binomial. Additionally, the survival model failed to converge, so we used an optimizer (‘nlminb’) from the package ‘optimx’ that solved the problem (Nash & Varadhan, 2011). For development time, the data had a right‐skewed distribution. Thus, we used log‐transformed data with residuals that had a normal distribution. All data were analyzed in R environment R.4.3.2 (R Core Team, 2021) using the function ‘lmer’ or ‘glmer’ from the package ‘lme4’ for the LMMs (Bates et al., 2015). We used the function ‘Anova’ to obtain the summary statistics for each model. Models were checked for assumptions using the function ‘check_model’ from the package ‘performance’ (Lüdecke et al., 2021). We used the package ‘emmeans’ to evaluate pairwise comparisons for each model (Lenth et al., 2018), focusing only on pairwise comparisons of parental hosts within each larval host plant treatment.

## RESULTS

We found a significant interaction between larval host plant and parental host plant for survival rate (χ^2^ = 20.5, df = 9, *p* = 0.015; Figure 2A), pupal mass (χ^2^ = 35.23, df = 9, *p* < 0.0001; Figure 2B), and development time (χ^2^ = 76.65, df = 9, *p* < 0.0001; Figure 2C). When larvae fed on willow, survival was lower when parents fed on alder compared to willow. When larvae fed on alder, pupal mass was lower when parents fed on cherry compared to larvae from parents fed on narrowleaf, and development time was shorter for larvae from parents fed on narrowleaf. When larvae fed narrowleaf, they had shorter development time when the parents also fed on narrowleaf compared to parents fed on alder.

**FIGURE 2:**
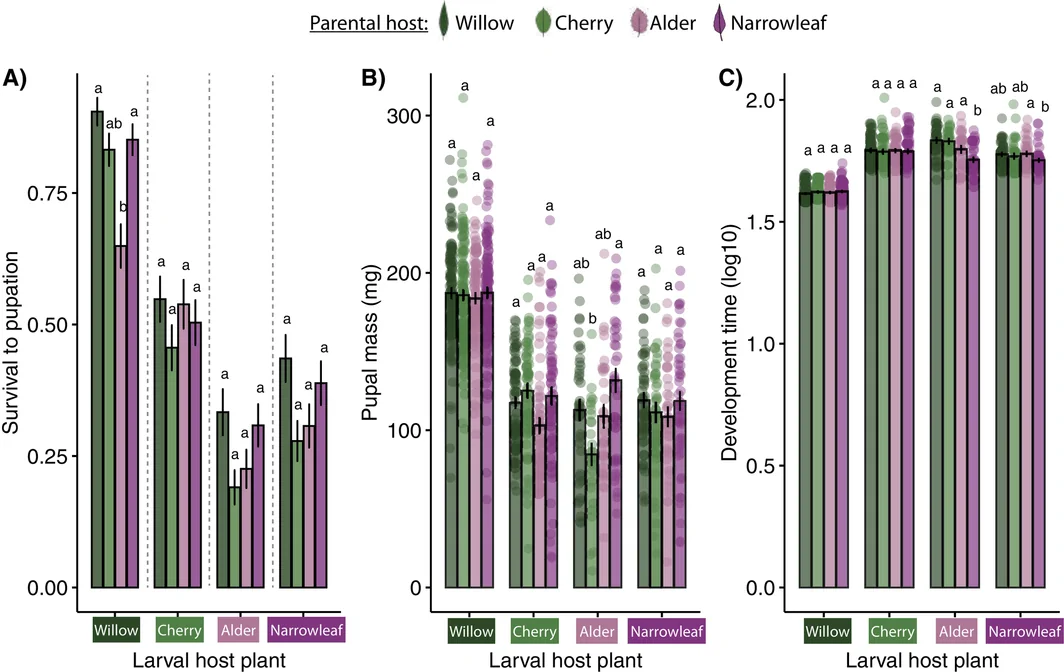
Performance of larvae on each host plant according to their parental host plant, measured as (A) proportion of survival, (B) pupal mass, and (C) development time. Bars represent mean ± SE and treatments with the same letter(s) above the bars are not significantly different. Pairwise comparisons are done only within the same larval host plant.

Larval host plant was also significant for all performance metrics (survival: χ^2^ = 321.14, df = 3, *p* < 0.0001; pupal mass: χ^2^ = 1195.5, df = 3, *p* < 0.0001; development time: χ^2^ = 3826.01, df = 3, *p* < 0.0001), with larvae overall performing better on willow than any other host plant (Figure 2). Parental host plant was not significant (survival: χ^2^ = 4.69, df = 3, *p* = 0.195; pupal mass: χ^2^ = 1.35, df = 3, *p* = 0.716; development time: χ^2^ = 1.65, df = 3, *p* = 0.649). Lastly, sex was also significant for pupal mass (χ^2^ = 129.23, df = 1, *p* < 0.0001).

## DISCUSSION

We found that parental effects played a limited and inconsistent role in the performance of a dietary generalist herbivore, but this lack of a consistent pattern may help explain the maintenance of dietary generalism. We found that parent‐offspring host matching never improved offspring fitness compared to most of the mismatched larvae reared on that same host plant, and the negative effects of host mismatching were limited. When analyzing performance by larval host plants, we found an interaction between larval host and parental host, such that larvae from parents that fed on a high‐quality host (willow) had higher survival on that host relative to larvae from parents fed on alder. Consistent with this pattern, studies of the polyphagous moth *Spodoptera littoralis* have also found support for a benefit of host matching for high‐quality hosts (Rösvik et al., 2020). For hosts other than willow, responses were heterogeneous, and there were no consistent positive or negative effects of parent‐offspring match or mismatch. Thus, our results suggest there is no benefit for FW as a generalist to specialize, and indeed specializing on most of the host plants in our study would not be adaptive.

The negative effects of host mismatch on fitness appear to result solely from the lower quality of the alternative host plants (alder). Offspring from willow‐fed parents performed similarly on other plants compared to host‐matching larvae of other hosts, but not better, indicating no evidence of a silver spoon effect associated with this high‐quality host plant. In contrast, when parental host mismatch involved offspring feeding on willow but originating from parents reared on other hosts, most larvae performed well regardless of parental host environments relative to matched willow offspring from willow parents. The exception was larvae from alder‐fed parents, which had lower survival on willow compared to larvae from willow‐fed parents or narrowleaf‐fed parents; however, these offspring still outperformed their siblings reared on alder. Thus, the negative effects of parent‐offspring host mismatch were not consistent across hosts. When parents from alder, cherry, and narrowleaf had offspring that were fed on willow (parent‐host mismatch), those offspring performed better than larvae from alder, cherry, and narrowleaf that matched their relative parental hosts. Thus, the consequences of parent‐offspring host match versus mismatch seem to depend mostly on whether the individual is feeding on willow. However, willow does not appear to be a ‘silver‐spoon’ host plant because having a willow‐fed parent does not confer a fitness advantage to offspring when fed any of the host plants (parent‐offspring match or mismatch).

The lack of a ‘silver‐spoon’ parental effect in our system may be because FW do not seem to increase allocation of resources to their offspring when reared on high‐quality host plants (Sellers, Hicks, et al., 2026). In other herbivorous insects, one potential mechanism for beneficial parental effects is that individuals feeding on high‐quality plants allocate greater resources to their offspring, leading to increased larval performance (Braby, 1994; Delisle & Bouchard, 1995; Fox et al., 1995; Levin et al., 2017; Muller et al., 2016). Additionally, egg size appears to be particularly important, as Rossiter (1991) found that offspring that hatched from larger eggs developed faster and had larger pupal mass, suggesting this maternal provisioning can affect offspring fitness. However, in our previous study testing female FW egg cluster size and egg lipid provisioning, we found no increase in lipid allocation or egg cluster size when females were fed a higher quality host plant (Sellers, Hicks, et al., 2026). Other than direct egg provisioning, females could provide parental effects through host plant choice (Cahenzli & Erhardt, 2013). Notably, we have found no evidence in FW of female choice or host preferences (Sellers et al., 2025).

For this study, we cannot distinguish between maternal and paternal effects because we reared both parents on the same hosts, but given that there are no significant overall parental effects, it seems unlikely that there would be selection for either maternal or paternal effects individually. Yet, other studies have found that host plant use by male parents can affect provisioning of their offspring through nuptial gifts (Boggs & Gilbert, 1979). Male butterflies can transfer nutrients to females during mating via the spermatophore (Wiklund et al., 1993); in some species, such as *Pieris napi*, receiving larger amounts of resources from males increased female reproductive effort (Wedell & Karlsson, 2003). Host plant quality can affect lifetime production of spermatophores (Muller et al., 2016) and spermatophore size (Delisle & Bouchard, 1995), which in turn can affect offspring provisioning. Further, the experiences of past generations, even grandparents, have been shown to affect offspring fitness (Wylde et al., 2019); our experiment did not include the diet of the grandparental generation in the experiment design, so we cannot assess grandparental effects. However, the lack of clear advantages due to parental effects suggests that, similarly to female provisioning, male provisioning and grandparent effects likely do not play a significant role in offspring performance.

In our study, with an extreme generalist that feeds on over 600 plant species worldwide (Schowalter & Ring, 2017; Warren & Tádic, 1970), we did not find clear evidence of parental effects. Parental effects are similarly absent in other generalists, such as soft scale insects that feed on 296 plant species across 84 families (Spitzer, 2004). Furthermore, parental effects are not always consistently found in specialists; parental effects occur in some specialists, such as seed‐feeding bruchid beetles (Amarillo‐Suárez & Fox, 2006; Or & Ward, 2007), but not in other specialists, such as pea aphids and milkweed bugs (McLean et al., 2009; Newcombe et al., 2015; Via, 1991). Similar to our study, diet quality played a larger role than maternal effects in specialist milkweed bugs (Newcombe et al., 2015). Moreover, while maternal provisioning is thought to be a mechanism that facilitates switching to a novel host plant, previous studies on specialists (Newcombe et al., 2015) and our own study on an extreme generalist do not support this notion. While parental effects may be beneficial under some circumstances, they may be limiting in others. If an herbivore is always constrained by the environment their parents experience, this may limit their own ability to adjust to their host plant. Particularly for an extreme generalist like FW, plasticity at the level of the individual may facilitate their ability to feed on a variety of different host plants.

To date, studies of transgenerational parental effects on herbivore diet breadth—including our own—have largely been system‐specific, lacking cross‐system synthesis, which has allowed conflicting hypotheses to persist regarding the ecological conditions under which anticipatory parental effects are most likely to evolve (Lind et al., 2020). While such effects are thought to prime offspring for locally predictable environments (e.g., abundant host plants), it remains unclear whether these benefits are most pronounced in dietary specialists, which experience predictable environments, or in generalists, which may face greater local host variability (Rösvik et al., 2020). As a future direction, a meta‐analysis would provide a quantitative comparison of the strength and direction of parental effects across phytophagous insect species, explicitly testing how these effects vary along the specialist‐generalist continuum. Such a synthesis would help resolve outstanding uncertainty in the field and provide a conceptual framework for future comparative research on transgenerational plasticity.

We found that parental effects did not consistently impact FW performance, and that the single parental effect we found was context dependent, relying more on host plant identity than host matching or mismatching. If parent‐offspring match does not often provide any performance benefit to offspring, this may help maintain dietary generalism when there is no selection for offspring host fidelity. Even though we found that willow is in general a high‐quality host, we might not see specialization to this host due to other factors such as lack of oviposition preference and top‐down effects. Our study only considered bottom‐up effects, and there might be other disadvantages not considered here. For instance, our previous research found lower parasitism rates on alder, which is a low‐quality host when only bottom‐up effects are considered, compared to higher quality hosts (Murphy & Loewy, 2015). Ultimately, we did not find a benefit of parent‐offspring host matching in this extreme dietary generalist, and the absence of a parental effect may help explain the maintenance of generalism in a world of specialists.

## AUTHOR CONTRIBUTIONS

Shannon M. Murphy and Emma J. Sellers conceived of the study. Emma J. Sellers, Shannon M. Murphy, Mykaela Tanino‐Springsteen, and Dhaval K. Vyas conducted the experiment. Mayra C. Vidal and Gina M. Wimp conducted the statistical analyses. All authors worked together to write and edit the manuscript. All authors read and approved the final manuscript.

## CONFLICT OF INTEREST STATEMENT

The authors declare no conflicts of interest.

## Supporting information


Appendix S1.

## Data Availability

Data (Sellers, Tanino‐Springsteen, et al., 2026) are available in Dryad at https://doi.org/10.5061/dryad.j3tx95xr0.
