# Supplementary material for: Parents don't dictate the menu: Lack of strong host‐specific parental effects may help maintain dietary generalism
Source: Ecology. 2026 Oct 6;107(10):e70499. doi: 10.1002/ecy.70499 (PMC13639905; doi:10.1002/ecy.70499)
Supplement: Supplementary file 1 [file ECY-107-e70499-s001.pdf]

Appendix S1

Parents don’t dictate the menu: Lack of strong host-specific parental effects may help maintain dietary generalism

Emma J. Sellers, Mayra C. Vidal, Dhaval K. Vyas, Mykaela Tanino-Springsteen, Gina M. Wimp, Mattheau S. Comerford, Mariana Abarca, Shannon M. Murphy

Ecology

Table S1. Number of larvae reared on each host plant from each parental host plant treatment. The parental hosts are listed with the number of matrilines created on each parental host listed below, with a total of 38 matrilines. The larval hosts are listed by rows, which give the total number of larvae reared from each parental host plant on each larval host plant. Totals across larval hosts within a parental host are given at the bottom of each column.

Table S1

Parental Host	Larvae per Larval Host	Total larvae per Parental Host
willow (*Salix nigra*) Matrilines # 1-9	willow: n = 126	n = 502 larvae
	cherry: n = 135	
	alder: n = 117	
	narrowleaf: n = 124	
cherry (*Prunus virginiana*) Matrilines # 10-19	willow: n = 149	n = 572 larvae
	cherry: n = 136	
	alder: n = 147	
	narrowleaf: n = 140	
alder (*Alnus tenuifolia*) Matrilines # 20-28	willow: n = 134	n = 511 larvae
	cherry: n = 117	
	alder: n = 133	
	narrowleaf: n = 127	
narrowleaf (*Populus augustifolia*) Matrilines # 29-38	willow: n = 148	n = 559 larvae
	cherry: n = 139	
	alder: n = 133	
	narrowleaf: n = 139	
total matrilines: n = 38	total experimental larvae: n = 2144	
